# Population genetic structure, differentiation, and diversity in *Tetrix subulata* pygmy grasshoppers: roles of population size and immigration

**DOI:** 10.1002/ece3.2520

**Published:** 2016-10-09

**Authors:** Jon Tinnert, Olof Hellgren, Jenny Lindberg, Per Koch‐Schmidt, Anders Forsman

**Affiliations:** ^1^ Department of Biology and Environmental Science Ecology and Evolution in Microbial Model Systems, EEMIS Linnaeus University Kalmar Sweden; ^2^ Present address: Department of Biology Lund University Lund Sweden; ^3^ Present address: Naturbruksskolan Sötåsen 54591 Töreboda Sweden

**Keywords:** dispersal, evolution, gene flow, Orthoptera, polymorphism, population divergence, *Tetrix subulata*

## Abstract

Genetic diversity within and among populations and species is influenced by complex demographic and evolutionary processes. Despite extensive research, there is no consensus regarding how landscape structure, spatial distribution, gene flow, and population dynamics impact genetic composition of natural populations. Here, we used amplified fragment length polymorphisms (AFLPs) to investigate effects of population size, geographic isolation, immigration, and gene flow on genetic structure, divergence, and diversity in populations of *Tetrix subulata* pygmy grasshoppers (Orthoptera: Tetrigidae) from 20 sampling locations in southern Sweden. Analyses of 1564 AFLP markers revealed low to moderate levels of genetic diversity (PPL = 59.5–90.1; Hj = 0.23–0.32) within and significant divergence among sampling localities. This suggests that evolution of functional traits in response to divergent selection is possible and that gene flow is restricted. Genetic diversity increased with population size and with increasing proportion of long‐winged phenotypes (a proxy of recent immigration) across populations on the island of Öland, but not on the mainland. Our data further suggested that the open water separating Öland from the mainland acts as a dispersal barrier that restricts migration and leads to genetic divergence among regions. Isolation by distance was evident for short interpopulation distances on the mainland, but gradually disappeared as populations separated by longer distances were included. Results illustrate that integrating ecological and molecular data is key to identifying drivers of population genetic structure in natural populations. Our findings also underscore the importance of landscape structure and spatial sampling scheme for conclusions regarding the role of gene flow and isolation by distance.

## 1 Introduction

The patterns of genetic diversity seen within and among populations and species are influenced by a complex interplay of ecological and evolutionary processes. It has long been recognized that the effects of stochastic events and selection depend on the spatial and temporal scales of environmental variation relative to the mobility, behavior, dispersal capacity, and life span of the organism (Baguette & Van Dyck, 2007; Bell, 2010; Ellner, 1996; Frank & Slatkin, 1990; Haldane & Jayakar, 1963; Hanski, 1998; Hedrick, 1986, 2006; Levins, 1968; Roff, 1992, 1997; Seger & Brockmann, 1987). Despite extensive research, however, there remain many unresolved issues.

It is commonly stated that dispersal and gene flow typically has a homogenizing effect, which dilutes genetic differences between populations. Isolation by distance (IBD) describes the accumulation of local genetic differences under geographically restricted dispersal, and an expectation under the IBD hypothesis is that neutral genetic differentiation will increase with increasing geographic distance (Slatkin, 1993; Wright, 1943). However, evaluation of this hypothesis is difficult because signatures of IBD depend on the spatial scale of the sampling regime in relation to the dispersal capacity of the study organism, landscape characteristics, and the arrangement of suitable habitat patches throughout the study area (Merimans, 2012; van Strien, Holderegger, & Van Heck, 2015; Yang, Novembre, Eskin, & Halperin, 2012). Moreover, dispersal and gene flow may be asymmetric, typically occurring at higher rates from larger and more productive populations to smaller populations (Fraser, Lippé, & Bernatchez, 2004; Hanski, 1998; Lande, 1988). In addition, gene flow, genetic structure, and population differentiation can be affected by landscape features and human land use, which might modify connectivity patterns and constitute partial or complete barriers to dispersal (Alcala, Streit, Goudet, & Vuilleumier, 2013; Jha, 2015; Mager, Colson, Groves, & Hundertmark, 2014; Noguerales, García‐Navas, Cordero, & Ortego, 2016; Ruiz‐Gonzalez, Cushman, Madeira, Randi, & Gómez‐Moliner, 2015).

Evaluating how dispersal influences population structure is complicated further by the fact that dispersal does not always translate into gene flow. This is because immigrants may die before they reproduce, behavioral differences between individuals from different populations may function as mating barriers, and out‐breeding depression associated with admixture of genotypes from different locally adapted populations may result in nonviable offspring (Rius & Darling, 2014). Additionally, there is a growing awareness that dispersal may be nonrandom and that (instead of having a homogenizing effect) it may contribute to population divergence and promote the evolution of local adaptations (Clobert, Danchin, Dhondt, & Nichols, 2001; Edelaar & Bolnick, 2012; Edelaar, Siepielski, & Clobert, 2008; Hanski, 1998; Karpestam, Wennersten, & Forsman, 2012; Phillips, Brown, Webb, & Shine, 2006; Reznick & Ghalambor, 2001). The role of immigration for genetic structure can potentially be evaluated by examining whether genetic differentiation between populations or differences among populations in the level of within‐population genetic diversity are associated with variation in the incidence of dispersive or immigrant phenotypes (Peterson & Denno, 1997), but few study systems are suitable for such investigations.

With regard to the effect of population dynamics on genetic structure, diversity, and differentiation, it is generally expected that small population size, bottlenecks, and founder events generally result in losses of alleles and low genetic diversity within populations (Frankham, 1996). Yet, many studies have reported surprisingly high levels of genetic variation in small and peripheral populations (Simberloff, 2009). In some cases, this may be related to positive effects of founder genetic diversity on establishment (Forsman, 2014), but the reasons for the surprisingly high levels of diversity are often unclear. Whereas large populations are expected to be genetically more diverse, small population size can contribute to greater genetic differentiation between populations due to drift (Mager et al., 2014). However, the influence of population size on genetic distance has seldom been considered.

**Figure 1:**
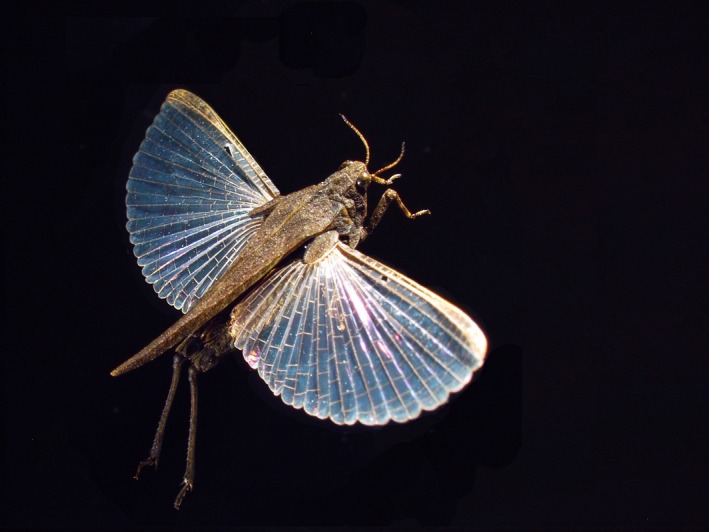
A *Tetrix subulata* pygmy grasshopper female belonging to the macropterous morph with long and functional wings. Photograph: J. Tinnert

A deeper understanding and resolution of the above issues requires that the fundamental ecological and evolutionary processes are studied in a variety of model systems. *Tetrix subulata* (L) pygmy grasshoppers (Orthoptera, Tetrigidae) (Figure 1) are suitable for investigating effects of landscape structure, geographic isolation, immigration, and population size on genetic structure, divergence, and diversity of populations. Pygmy grasshoppers provide a classic model system for studies of color polymorphism (Forsman, Karlsson, Wennersten, Johansson, & Karpestam, 2011; Nabours, 1929), and they occupy a broad range of habitat types. *T. subulata* usually occur in low densities but have a high reproductive capacity and may rapidly become very numerous when and where conditions are favorable (Forsman et al., 2011). As such, they share characteristics akin to “ruderal species” of plants, in that they thrive in habitats disturbed by fires, cultivation, trampling by cattle, or wave action and seem to use an environmental tracking strategy.

Mark–recapture data of free‐ranging individuals indicate that *T. subulata* normally move only a few meters per day (Berggren, Tinnert, & Forsman, 2012; Caesar, Ahnesjö, & Forsman, 2007; Forsman & Appelqvist, 1999). However, *T. subulata* is wing dimorphic (Nabours, 1929; Rehn & Grant, 1955), and macropterous individuals with functional wings (Figure 1) may resort to active flights of up to 75 m, thus indicating a capacity for long‐distance dispersal (Berggren et al., 2012). The long‐winged morph is more common in disturbed than in stable habitats, and it consistently declines in frequency over time within newly founded populations that inhabit disturbed and changing habitats (Berggren et al., 2012).

Pygmy grasshoppers are promiscuous (Caesar & Forsman, 2009), and female *T. subulata* mated to several males may produce offspring that are half‐siblings which are phenotypically and genetically more diverse (Forsman, Ahnesjö, & Caesar, 2007; Johansson, Caesar, & Forsman, 2013). Because females produce half‐sibling offspring, a few founder females that have mated with multiple males may potentially give rise to new populations that comprise much of the genetic variation in the source population (Johansson et al., 2013; Pearse & Anderson, 2009). Accordingly, there is experimental evidence that new populations may be established by small founder groups of only six individuals (Forsman, Wennersten, Karlsson, & Caesar, 2012; see also Wennersten, Johansson, Karpestam, & Forsman, 2012). Taken together, this suggests that a high incidence of the long‐winged flight‐capable morph may be used as a proxy to identify *T. subulata* populations either that have been recently established (and hence might be hypothesized to show signatures associated with founder events), or that represent older populations that have been much influenced by recent immigration (and that might show signatures associated with admixture).

The ecological characteristics and transient (metapopulation) population dynamics make pygmy grasshoppers an interesting model system for studies of population genetic structure. Our goals in this study were to use amplified fragment length polymorphism (AFLP) data (Bensch & Åkesson, 2005; Vos et al., 1995) to investigate genetic structure, population differentiation, and genetic diversity in relation to immigration (as estimated by the incidence of long‐winged individuals), population size, landscape features, and physical isolation in 20 *T. subulata* pygmy grasshopper populations in southern Sweden including the island of Öland.

We hypothesized that there is significant genetic divergence between mainland and island populations and that the genetic distance between populations increases with geographic distance within the two regions, due to restricted dispersal and gene flow. Population size was expected to influence genetic diversity within populations as well as the rate of genetic divergence between populations, due to drift. The expectation regarding the influence of dispersive phenotypes on genetic structure is more complicated: genetic diversity was expected to increase with increasing proportion of long‐winged phenotypes under the assumption that it represented higher rates of immigration and gene flow, whereas genetic diversity was predicted to be lower if populations with a high incidence of long‐winged phenotypes represent newly established populations, due to founder effects. The influence of immigration on genetic diversity was also predicted to differ between mainland and island populations, due to differences in land use and connectivity.

## 2 Material and Methods

### 2.1 Study species

*Tetrix subulata* is an Orthopteran of the Tetrigidae family. It is a small (<15 mm total body length, mean 0.07 g dry body mass), diurnal, ground‐dwelling, and widely distributed insect that inhabits biomes ranging from tropical rainforests to arctic regions of Europe, Asia, and much of North America south to Mexico (Holst, 1986; Rehn & Grant, 1955). It usually occupies damper microhabitats in relatively open areas (e.g., clear cuttings, shore meadows, pastures), where it lives on the soil surface and feeds on microalgae growing on moist soils, mosses, and detritus (Holst, 1986; Karpestam & Forsman, 2011). Adult and late instars nymphs hibernate during winter and emerge in April–May when reproduction ensues. Females survive at most one reproductive season and produce multiple pods of egg (<35 eggs/clutch), and nymphs develop through five (males) or six (females) instars before enclosing.

### 2.2 Sampling and study area

**Figure 2:**
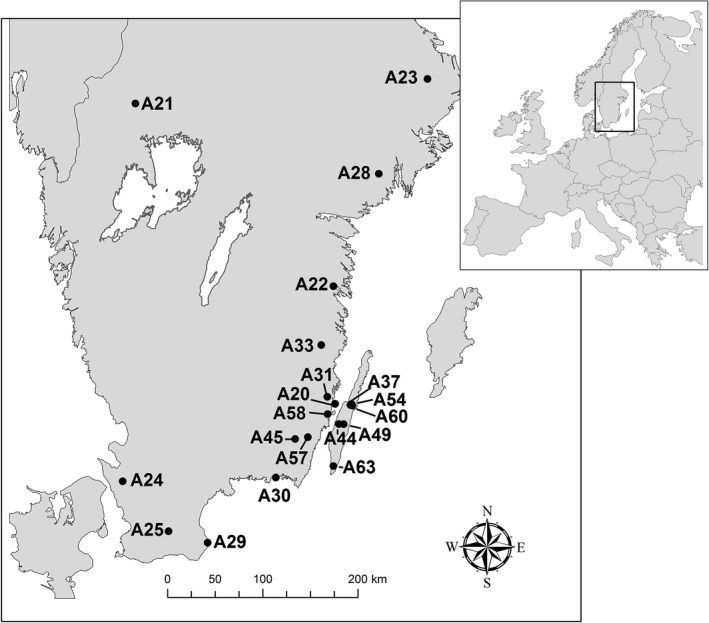
Map of study area in the south of Sweden, showing sample ID of 20 sampled locations of *Tetrix subulata* pygmy grasshoppers. See Table 1 for a key to abbreviations of sampling locations

During 2007–2012, we collected *T. subulata* pygmy grasshoppers from 20 natural populations in Sweden (Table 1, Figure 2). We selected sampling localities on the Swedish mainland and on the island of Öland, situated off the Swedish east coast in the Baltic Sea, such that our data set contained populations with varying degrees of interpopulation distances and connectedness. The sampling localities represented rather similar types of habitats, including a stream shoreline, clear‐cut areas, pastures, and a meadow (Table 1). Grasshoppers were collected in spring and early summer (for details, see below and Forsman et al., 2011, 2012). Captured individuals were identified to species according to Holst (1986), classified according to sex and wing morph (Berggren et al., 2012), and preserved in 90% ethanol until DNA extraction. The number of individuals used for AFLP analyses is sometimes different from the total number of collected individuals and the sample sizes used to calculate the proportion of long‐winged individuals (Table 1). These discrepancies arose because some of the collected individuals were nymphs, which can be used for AFLP analyses but not for classification of wing morph. For some of the locations, only a subsample of the collected individuals was brought to the laboratory for classification of wing morph and DNA extraction, whereas remaining individuals were released at the sampling location.

**Table 1:** Genetic diversity in samples of *Tetrix subulata* pygmy grasshoppers collected from 20 sampling locations on the Swedish mainland and on the island of Öland off the Swedish east coast in the Baltic Sea. *N* indicates number of sampled individuals used for AFLP analyses, PL indicates number of polymorphic loci, PPL indicates percentage polymorphic loci, and Hj indicates genetic diversity. Collected indicates number of individuals collected during one visit. Long‐winged indicates the proportion of phenotypes with functional wings in a sample of (*N*) adult individuals

Sample ID	Region	Location	Year	*N*	Habitat type	Collected	Long‐winged (*N*)	Coordinates Lat. Long.	PL	PPL	Hj (SE)
A20	Mainland	Eneskärskläppen	2011	11	Island vegetation	16	1 (16)	56.866850°, 16.495717°	1077	68.8	0.23658 (0.00465)
A21	Mainland	Edane	2012	14	Pasture	22	0.86 (22)	59.667833°, 12.830050°	1270	81.2	0.26460 (0.00439)
A22	Mainland	Tindered	2012	8	Pasture	10	1 (10)	57.977083°, 16.484550°	1028	65.7	0.24035 (0.00470)
A23	Mainland	Knutby	2012	21	Pasture	23	0.65 (20)	59.909033°, 18.290467°	1079	68.9	0.23973 (0.00457)
A24	Mainland	Hyllinge	2012	22	Pond	35	0.67 (35)	56.103217°, 12.895167°	1110	70.9	0.24937 (0.00458)
A25	Mainland	Sjöbo	2012	25	Pasture	45	0.67 (45)	55.648617°, 13.694067°	1032	65.9	0.24289 (0.00459)
A28	Mainland	Gnesta	2012	24	Wet agricultural area	34	0.8 (30)	59.029900°, 17.335900°	1020	65.2	0.24301 (0.00458)
A29	Mainland	Simrishamn	2012	26	Pasture	26	0 (24)	55.549767°, 14.351933°	1035	66.1	0.24260 (0.00458)
A30	Mainland	Tomtesunda	2012	9	Pasture	9	1 (9)	56.173883°, 15.479167°	1064	68.0	0.24491 (0.00469)
A31	Mainland	Ålem	2009	16	Agricultural area	278	0.91 (11)	56.933650°, 16.363467°	1127	72.0	0.28354 (0.00457)
A33	Mainland	Hägern	2009	15	Meadow nearby burnt area	104	0.93 (15)	57.423067°, 16.266067°	1092	69.8	0.27692 (0.00455)
A45	Mainland	Sävsjö	2011	22	Pasture with pond	295	0.97 (198)	56.537800°, 15.803867°	1299	83.0	0.30501 (0.00430)
A57	Mainland	Aspelund	2008	4	Pasture with stream	46	0.25 (4)	56.553767°, 16.022617°	931	59.5	0.27526 (0.00462)
A58	Mainland	Björnö	2008	9	Pasture	51		56.770617°, 16.364550°	1145	73.2	0.27896 (0.00479)
A37	Öland	Bredsätra	2011	24	Pasture with pond	52	0.12 (52)	56.849283°, 16.788883°	982	62.7	0.24704 (0.00476)
A44	Öland	Vanserumbäck	2011	19	Pasture with stream	243	0.3 (200)	56.673733°, 16.636900°	1321	84.4	0.31653 (0.00416)
A49	Öland	Jordtorp	2011	21	Pasture and alkaline fen	36	0.17 (36)	56.676883°, 16.555583°	1050	67.1	0.23756 (0.00469)
A54	Öland	Norra mossen	2007	17	Pasture	31	0.4 (25)	56.861850°, 16.779167°	1290	82.4	0.30970 (0.00410)
A60	Öland	Hörninge	2008	16	Clear‐cut with alkaline fen	28	0.74 (27)	56.858100°, 16.768433°	1410	90.1	0.31863 (0.00416)
A63	Öland	Lindby	2010	20	Alkaline grassland with stream	137	0.08 (131)	56.280283°, 16.456100°	1111	71.0	0.25635 (0.00463)
		Total		343		1,548			1,564		

The number of grasshopper individuals collected at each locality during one visit was recorded and used as a proxy for population size. The number of collected individuals at each site underestimates actual population size, but it is a reliable relative measure and robust to factors that could potentially influence total catch, such as differences in the area covered for sampling, time invested in sampling, number of people involved in each sampling event, habitat type, and weather conditions. We searched for grasshoppers while walking slowly through the area during days with weather conditions suitable for grasshopper activity, that is, clear or overcast days with a temperature of at least 15°C (Forsman et al., 2011, 2012). *Tetrix subulata* does not have a uniform or random spatial distribution (Ahnesjö & Forsman, 2006; Forsman & Appelqvist, 1999), and we therefore initially searched the entire areas and then concentrated our search and capture effort to those parts, microhabitats, and substrate types (i.e., humid bare soil, areas covered by mosses) that are preferred by pygmy grasshoppers (Ahnesjö & Forsman, 2006; Berggren et al., 2012; Forsman et al., 2011, 2012). Because *T. subulata* predominantly move around on the ground surface and rarely climb vegetation, they are difficult to capture with a bag net, and we therefore acted like visual predators and captured by hand individuals that we could see (Forsman & Appelqvist, 1999; Forsman et al., 2012).

Capture probability is similar across studies of *T. subulata* despite varying conditions. In a previous capture–mark–recapture study (carried out between 8 May and 26 June, 1996, in a 4‐year‐old clear‐cut field that had been ravaged by fire), in which 442 marked *T. subulata* were released and recaptured by two people on five occasions, capture probability (40%) did not vary over time periods despite that weather conditions were different (Forsman & Appelqvist, 1999). One year later, on 5 July, 1997, the same researchers marked and released 196 individuals at the same site to study movement patterns, and 73 (37%) of these were recaptured 4 days later (Forsman & Appelqvist, 1999). In a more recent study (Berggren et al., 2012), 73 marked *T. subulata* were released in a cattle‐grazed pasture on 27 May, 2010, and 28 (38%) of these were recaptured 4 days later when the area was searched by three people.

Humans that search for pygmy grasshoppers tend to behave in accordance with optimal foraging theory. The cumulative number of grasshoppers collected increases asymptotically with search time. When several people search at the same area, the asymptote is reached faster but the cumulative number of grasshoppers collected does not increase. This is likely because the number and density of grasshoppers remaining in an area decreases in an exponential decaying manner over time, and individuals tend to stop searching when detection rate drops below a critical level. Thus, giving up density is almost invariable, but giving up time decreases when more people are searching, such that total search effort is comparable across sampling sites and/or occasions. For subsets of the 20 sampling locations, we recorded the number of people involved (*n *=* *19 locations) and search time (*n *=* *17 locations) in addition to the total number of grasshoppers collected. Total number of grasshoppers collected increased with increasing number of people involved in the search (*r*_*s*_ = .77, *n *=* *19, *p *<* *.0001) but was not significantly associated with search time (*r*_*s*_ = .47, *n *=* *17, *p *=* *.054). It also was not necessary to adjust for search time because the questions addressed concerned population size rather than population density. There was a strong correlation between total number of grasshoppers collected and number of grasshoppers collected per person involved in the search (*r *=* *.94, *n *=* *19, *p *<* *.0001), indicating that total number of grasshoppers collected is a reliable surrogate for population size.

To enable inclusion of data for all sampling locations, results reported below were obtained using total number of grasshoppers collected as a proxy for population size. However, results regarding the associations of genetic diversity (Hj) with population size remained qualitatively unchanged when we used the restricted data set and treated number of grasshoppers collected per person as a proxy for population size to control for differences in search effort.

The proportion of long‐winged individuals at each site was used as a proxy for immigration rate. Earlier work (Berggren et al., 2012) has shown that the proportion of males and females that belong to the macropterous long‐winged morph is highly correlated across samples from different populations and years [correlation on arcsine square‐root‐transformed proportions, *F*_1,18_ = 43.41, *r *=* *.84, *n *=* *20 samples, *p *<* *.0001, analysis based on data for 658 males and 1815 females reported in Berggren et al. (2012)], and that the incidence of the long‐winged morph does not differ consistently between males and females in this part of the distribution range (paired *t*‐test, *t *=* *0.27, *n *=* *20, *p *=* *.79). Furthermore, results from previous capture–mark–recapture studies show that capture probability is independent of both sex (Forsman & Appelqvist, 1999) and wing morph (Berggren et al., 2012). It is therefore unlikely that the estimates of the proportion of long‐winged phenotypes reported in this study (Table 1) were influenced to any important degree by any sampling bias according to sex or wing morph, or by any differences in sex ratio among samples from different collection sites. Furthermore, it is only the macropterous phenotype that is able to fly (Berggren et al., 2012). Taken together, this suggests that a high incidence of the long‐winged flight‐capable morph may be used as a proxy to identify *T. subulata* populations that have either been recently established (and hence might be hypothesized to show signatures associated with founder events), or that represent older populations that have been much influenced by recent immigration (and that might show signatures associated with admixture).

### 2.3 DNA extraction and molecular genetics analyses

From each of the 20 sampling locations, we used 4–26 (mean = 17) individuals for DNA extraction (Table 1) and DNA was extracted from the femur of each individual using the phenol–chloroform method according to Sambrook (Sambrook, Fritch, & Maniatis, 2002).

Analysis of AFLP was carried out as described by Vos (Bensch & Åkesson, 2005; Vos et al., 1995), using the restriction enzymes EcoRI and Tru1, the adapters EcoRI and MseI, the preamplification primers M_C_ X E_T_, and four combinations of selective primers (pair 1‐E_TAG_ X M_CGA_, pair 2‐E_TAG_ X M_CAG_, pair 3‐E_TCG_ X M_CAC_, and pair 4‐E_TAG_ X M_CAC_). Three negative controls and nine positive controls were included on each plate. PCR reactions were diluted 1:8 of which 2 μl was sent to Uppsala Genome Center for fragment analysis using capillary electrophoresis on an ABI3730XL DNA Analyzer (Applied Biosystems).

Chromatograms from all PCR plates were visually evaluated using GeneMarker 2.6.4 (SoftGenetics) and cleaned by removing chromatogram files with chromatograms of poor quality. Chromatograms were visualized and evaluated using GeneMapper 5.0 (Applied Biosystems), and peak heights from a total of 1,970 polymorphic sites were extracted for further analysis. Peak heights were normalized and converted to a binary presence–absence matrix of genotypes with a locus selection threshold of 200 and a phenotype calling threshold of 10 using AFLPscore (Whitlock, Hipperson, Mannarelli, Butlin, & Burke, 2008). In AFLPscore, 70 duplicated sample pairs were used to filter out and remove poor‐quality loci with high mismatch error rate. This resulted in a binary matrix of 1564 loci, consisting of ones and ceros. The results on genetic diversity and structure reported below are based on this large (1564 AFLP loci) matrix.

To assess the role of any genotyping errors, data for the nine positive control individuals from different collection sites that were replicated across all plates were used to measure genotyping repeatability, which was calculated for each allele using mismatch error rate (Bonin et al., 2004). Alleles with the highest mismatch error rate were iteratively removed until a smaller but higher quality data set with a low desired average error rate (mean: 4.8%, range 1.1%–14%, compared with an average error rate of 16.7% for the full data set) remained that consisted of only 638 loci. Results based on this reduced (638 loci) binary matrix were qualitatively similar to results based on the larger and more informative (1,564 loci) binary matrix reported below. The estimates and exact parameter values associated with the statistical tests changed somewhat, but overall conclusions were robust to choice of data matrix (for results based on the reduced (638 loci) matrix, see Supporting Results, Tables S1–S4, and Figs S1–S3).

All DNA extractions were performed in the same laboratory (Lund University, Lund), and our results and conclusions are therefore not influenced by any difficulties associated with transferring AFLP information across laboratories.

### 2.4 Estimates of genetic diversity within populations

Amplified fragment length polymorphism markers are dominant, and the estimation of allele frequencies was estimated from the proportion of recessive genotypes in the sample and performed using a Bayesian method with nonuniform prior distribution of allele frequencies, assuming Hardy–Weinberg proportions of genotypes and a predefined F_*IS*_ value of 0 (Zhivotovsky, 1999). Statistics of genetic diversity, number of polymorphic loci (PL), proportion of polymorphic loci (PPL) at the 5% level, and expected heterozygosity or Nei's genetic diversity Hj (analogous to He) (Nei, 1973), were estimated following the treatment of Lynch and Milligan (1994) using AFLPsurv (Vekemans, Beauwens, Lemaire, & Roldan‐Ruiz, 2002) and are reported in Table 1.

### 2.5 Analyses of genetic structure among populations

Genetic differentiation between sampling localities (pairwise *F*_ST_) was estimated using 5,000 permutations implemented in AFLPsurv (Vekemans et al., 2002). Gene flow between pairs of populations was calculated from these *F*_ST_ values, under the assumption of an infinite‐island model of population structure (Wright, 1951), based on the equation *Nm* = 0.25(1/*F*_ST_ − 1).

The island of Öland is situated 5 km off the coast of the Swedish mainland (Figure 2), and it can be hypothesized that the open water forms a dispersal barrier that influences population differentiation. To assess the impact of this potential barrier and evaluate genetic structure, we performed a nested analysis of molecular variance, AMOVA (Excoffier & Lischer, 2010). To this end, individuals were grouped by sampling site and then nested within two geographic regions (Öland and mainland). Genetic variation was estimated for the 1564 AFLP markers, and AMOVA was used to partition the proportion of total genetic variation explained: between geographic regions, among sample locations within regions, and among individuals within sample locations. The overall *F*_ST_ statistic, AMOVA, and Pairwise *F*_ST_ values were calculated as implemented in Arlequin version 3.5 (Excoffier & Lischer, 2010). The statistical significance of the AMOVA and the *F*_ST_ analyses was assessed by 1000 permutations of individuals among populations.

To evaluate the isolation‐by‐distance hypothesis (IBD), that is, whether the genetic differences between populations (as estimated by *F*_ST_) were correlated with the geographic distances (log km) that separated populations, we used a Mantel test with 10,000 randomizations implemented in ARLEQUIN version 3.5 (Excoffier & Lischer, 2010). Because the AMOVA results indicated a significant effect of geographic region, the IBD was evaluated both for the complete data set and for subsets of the data that comprised sample locations either from the Swedish mainland or from Öland.

Pairwise *F*_ST_ values may increase monotonically with increasing interpopulation geographic distances or increase up to a certain threshold distance beyond which the effects of gene flow can be so small compared with the effects of genetic drift and mutations that the expected *F*_ST_–distance correlation does not manifest anymore (Hutchison & Templeton, 1999; Rousset, 1997; van Strien et al., 2015). To further assess the presence and intensity of any isolation by distance in our data, we therefore evaluated the *F*_ST_–geographic distance correlation from subsets of mainland population pairs that differed with regard to threshold interpopulation distance (van Strien et al., 2015). For this analysis, we used five distance intervals (0–30, 0–50, 0–100, 0–250, and 0–550 km).

Those populations that were sampled at the northern, western, and southern outskirts of the mainland sampling area were relatively small (Table 1, Figure 2). Large populations are predicted to be genetically more diverse than small populations. There are also indications that population size can influence the genetic distance between populations (e.g., Mager et al., 2014). If rare alleles have been lost in smaller (and as it happened in more distantly located) populations due to drift or bottleneck effects, then this might generate a pattern where more distantly located populations would appear to be more closely related because they were small. To evaluate this potential bias, we investigated whether pairwise comparisons between two small (fewer than 30 collected individuals, Table 1) populations or between one large and one small population were similar to or generally generated lower pairwise *F*_ST_ values than comparisons between two large populations. Previous studies have used the harmonic mean population size to address this issue, but we consider it more appropriate to clearly separate population pairs consisting of one very small and one very large population from population pairs consisting of two intermediate‐sized populations.

To visualize the structure in the data, we first conducted a principal coordinate analysis (PCoA) using Jaccard distance, which calculates the dissimilarity between asymmetric binary variables, implemented in the vegan package from the software R Studio V.0.98.501 (R Development Core Team 2010), and then included all individuals in a biplot of the first and second PCoA axes.

Population genetic structuring in *T. subulata* was also investigated with a Bayesian clustering analysis implemented in STRUCTURE version 2.3.4 to test for significant patterns of clustering under a model assuming admixture and independent allele frequencies (Pritchard, Stephens, & Donnelly, 2000). Clustering number (*K*) ranged from 1 to 10, and each *K* was run 15 times using a burn‐in period of 10,000 iterations followed by 20,000 Markov chain Monte Carlo (MCMC) repetitions without using sample locations as local priors (Hubisz, Falush, Stephens, & Pritchard, 2009). The output results from STRUCTURE were analyzed to determine the number of clusters (*K*) that best fitted the distribution in the data using the Evanno method (Evanno, Regnaut, & Goudet, 2005), as implemented in Structure Harvester (Earl & vonHoldt, 2012). For individual assignment to populations, an independent run consisting of 5 million MCMC cycles and a burn‐in period of 50,000 was used, implementing the obtained optimal *K*.

### 2.6 Evaluating associations of within‐population genetic diversity with estimates of population size and immigration rate

We examined whether variation among populations in the degree of within‐population genetic diversity could be accounted for by differences in population size and immigration rate. Number of *T. subulata* collected per visit was used as a proxy for population size at each locality (mean = 77.7, range: 16–295 individuals, Table 1). Results were similar when number of grasshoppers collected per person was used as a proxy for population size, to control for differences in search effort. The proportion of long‐winged phenotypes at each site was used as a proxy for immigration rate (mean = 0.61, range: 0–1, Table 1). General linear model analysis of variance, implemented with procedure GLM in SAS, was used to test for effects on genetic diversity (Hj) of geographic region (mainland vs. island), population size (as estimated by number of individuals collected per visit), and immigration (as estimated by proportion of long‐winged individuals), and their interactions. We started with a fully saturated model that included all possible interactions between explanatory variables. Interactions that were not statistically significant (*p *>* *.10) were sequentially removed from the model, starting with the highest order three‐way interaction. Eta‐squared, η^2^, was calculated to estimate local effect size (Cohen, 1988). Population A58 was omitted due to missing data on wing morph frequency.

## 3 Results

### 3.1 Genetic diversity within populations

Our analyses based on 1,564 polymorphic AFLP markers for 343 *T. subulata* individuals collected from 20 sampling sites revealed high within‐population genetic diversity (proportion polymorphic loci and average heterozygosity) across all localities (PPL = 59.5–90.1; Hj = 0.24–0.32, Table 1).

### 3.2 Population genetic structure

The signature of genetic structure among populations was low to moderate, as indicated by pairwise *F*_ST_ values that ranged from 0 to 0.13 (AFLPsurv) (Table 2). Nearly, all (179 of 190) pairwise comparisons were statistically significant (Table 2).

**Table 2:** Population structure estimated in AFLPsurv with 1565 AFLP loci for 20 populations of *Tetrix subulata*. Lower matrix indicates *F*_ST_ values; upper matrix indicates *Nm* values. Pairwise comparisons shown in boldface indicate statistically significant (*p *<* *.05) *F*_ST_ values, as tested in ARLEQUIN. See Table 1 for a key to abbreviations of sampling locations. The dashed lines separate locations on the Swedish mainland from locations on the island of Öland

	A20	A21	A22	A23	A24	A25	A28	A29	A30	A31	A33	A45	A57	A58	A37	A44	A49	A54	A60	A63
A20		4.35	7.04	7.21	7.12	6.24	5.6	6.43	4.03	5.85	7.24	4.06	2.72	2.89	2.64	2.94	2.34	2.24	2.02	2.54
A21	**0.0544**		13.26	3.22	3.94	3.05	3.24	3.36	2.4	5.55	4.83	6.66	38.21	3.83	5.14	4.85	3.43	5.89	3.53	3.48
A22	**0.0343**	**0.0185**		5.38	6.1	4.53	5.52	5.1	2.89	4.48	4.24	3.64	6.51	2.57	4.47	2.91	4.04	3.28	2.21	3.9
A23	**0.0335**	**0.072**	**0.0444**		–	–	–	83.08	38.21	11.49	7.3	3.78	2.04	3.62	2.72	2.89	2.51	2.12	2.03	2.77
A24	**0.0339**	**0.0597**	**0.0394**	0		–	178.32	146.81	22.07	14.37	7.24	4.3	2.42	3.99	3.32	3.13	2.94	2.43	2.29	3.31
A25	**0.0385**	**0.0758**	**0.0523**	0	0		2499.75	75.51	21.3	11.43	7.51	3.94	2.02	3.92	2.74	2.96	2.52	2.14	2.16	2.87
A28	**0.0427**	**0.0716**	**0.0433**	0	0.0014	0.0001		131.33	38.21	12.57	6.4	3.52	2.08	3.34	2.79	2.85	2.67	2.18	2.05	3.04
A29	**0.0374**	**0.0693**	**0.0467**	0.003	0.0017	**0.0033**	0.0019		18	12.84	7.02	3.71	2.08	3.48	2.63	3.01	2.44	2.18	2.11	2.76
A30	**0.0584**	**0.0944**	**0.0796**	**0.0065**	**0.0112**	**0.0116**	**0.0065**	**0.0137**		7.04	4.39	2.9	1.66	2.7	2.31	2.26	2.27	1.81	1.73	2.47
A31	**0.041**	**0.0431**	**0.0528**	**0.0213**	**0.0171**	**0.0214**	**0.0195**	**0.0191**	**0.0343**		21.12	12.31	3.33	8.11	3.61	7.4	3.12	4.05	4.3	3.69
A33	**0.0334**	**0.0492**	**0.0557**	**0.0331**	**0.0334**	**0.0322**	**0.0376**	**0.0344**	**0.0539**	**0.0117**		38.81	3.13	10.25	2.68	12.51	2.15	3.19	4.6	2.77
A45	**0.058**	**0.0362**	**0.0642**	**0.0621**	**0.0549**	**0.0596**	**0.0664**	**0.0631**	**0.0793**	**0.0199**	**0.0064**		4.7	16.53	3.19	156	2.34	4.99	11.17	3.06
A57	**0.0842**	0.0065	**0.037**	**0.1093**	**0.0937**	**0.11**	**0.1074**	**0.1072**	**0.1308**	**0.0699**	**0.0739**	**0.0505**		2.81	9.4	3.66	4.88	13.64	3.04	5.05
A58	**0.0796**	**0.0613**	**0.0885**	**0.0646**	**0.0589**	**0.0599**	**0.0696**	**0.0671**	**0.0847**	**0.0299**	**0.0238**	**0.0149**	**0.0817**		2.57	10.08	1.92	3.13	13.26	2.51
A37	**0.0864**	**0.0464**	**0.053**	**0.0842**	**0.07**	**0.0837**	**0.0822**	**0.0867**	**0.0976**	**0.0647**	**0.0853**	**0.0727**	**0.0259**	**0.0887**		2.65	33.08	6.11	2.26	51.83
A44	**0.0783**	**0.049**	**0.079**	**0.0795**	**0.074**	**0.078**	**0.0807**	**0.0766**	**0.0997**	**0.0327**	**0.0196**	**0.0016**	**0.064**	**0.0242**	**0.0863**		2	4.67	17.23	2.59
A49	**0.0967**	**0.0679**	**0.0583**	**0.0906**	**0.0783**	**0.0902**	**0.0855**	**0.0931**	**0.0993**	**0.0742**	**0.1041**	**0.0966**	**0.0487**	**0.115**	**0.0075**	**0.1112**		3.9	1.76	25
A54	**0.1004**	**0.0407**	**0.0709**	**0.1056**	**0.0934**	**0.1044**	**0.1027**	**0.1027**	**0.1211**	**0.0582**	**0.0726**	**0.0477**	0.018	**0.0739**	**0.0393**	**0.0508**	**0.0602**		4.08	5.07
A60	**0.1099**	**0.0662**	**0.1015**	**0.1098**	**0.0984**	**0.1038**	**0.1088**	**0.1058**	**0.1261**	**0.0549**	**0.0515**	**0.0219**	**0.076**	**0.0185**	**0.0998**	**0.0143**	**0.1246**	**0.0578**		2.21
A63	**0.0895**	**0.0671**	**0.0603**	**0.0827**	**0.0703**	**0.0801**	**0.0761**	**0.083**	**0.0919**	**0.0634**	**0.0827**	**0.0756**	**0.0472**	**0.0907**	**0.0048**	**0.0881**	**0.0099**	**0.047**	**0.1018**	

**Figure 3:**
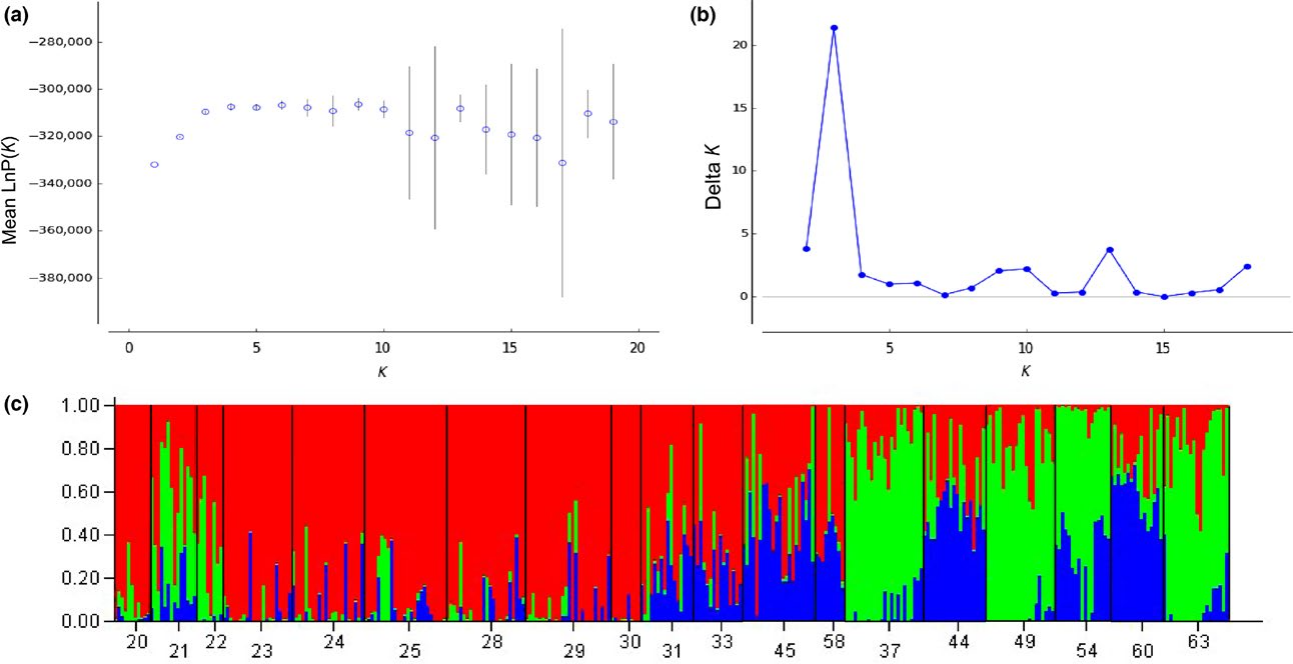
Bayesian population structure analysis used to assign 343 *Tetrix subulata* individuals to different genetic clusters for AFLP genotypes. (a) Mean likelihood of 10 simulations of 1–19 populations (*K*) of pygmy grasshoppers. (b) Change (delta *K*) in likelihood for *K* = 1–19 (Evanno et al., 2005). (c) Individual probability assignment of each of the individuals sampled in the 20 different locations for *K* = 3 populations. Individuals, sorted into the 20 sampling localities, are along the *x*‐axis. The *y*‐axis denotes the cumulative posterior probability of an individual's placement in particular population(s). Data based on 1,564 variable AFLP markers. See Table 1 for a key to abbreviations of sampling localities. Figure shows the result from a run with no prior information of sampling location

**Figure 4:**
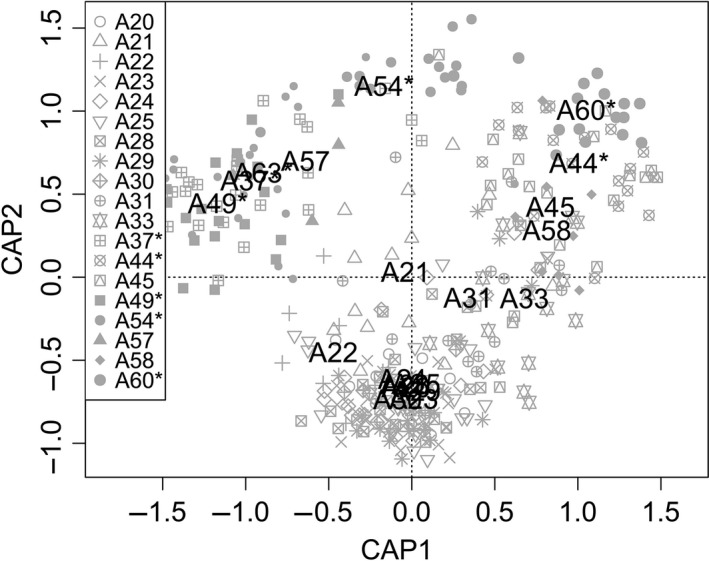
Principal Coordination analysis biplot for pygmy grasshopper *Tetrix subulata* individuals collected at 20 sampling locations for 1,564 AFLP markers calculated using Jaccard distance. Axis 1 (horizontal) accounted for 32.2% of the total variation, and axis 2 (vertical) accounted for 23.8% of the variation. *indicates samples from populations on the island of Öland (A37, A44, A49, A54, A60, and A63), located in the upper part of the plot

The likelihood values obtained in the assignment test (STRUCTURE) indicated that the individuals included in our data set clustered into three populations (*K *=* *3); the mean likelihood values reached a plateau after *K *=* *3 (Figure 3), and *K *=* *3 gave the highest delta *K* value (Evanno et al., 2005). Of the three genetic clusters, two represented localities from Öland and one represented localities from the Swedish mainland. The existence of three genetic clusters was illustrated by the results of the principal coordinate analysis (Figure 4), with the first and second PCoA axes accounting for 31% and 28% of the total variation in the data, respectively.

Results of the nested AMOVA provided statistically significant evidence for spatial population structure (Table 3). Nesting sample location by geographic region accounted for 4.7% of the total variance, while 7.2% was explained by variation among sample locations within regions, and 88.1% was explained by variation among individuals within sample locations. The genetic structure by region is illustrated in the PCoA biplot, in which all populations on Öland cluster in the upper part of the graph (Figure 4). Significant genetic structure by region was evident also when the mainland sample was restricted to the six populations (A20, A30, A31, A45, A57, and A58, see Figure 2) that were closest to the island of Öland (region (mainland vs. island) accounted for 2.3% of the total variance, *F*_ST_ = 0.12, *p *<* *.001).

**Table 3:** Genetic variance among geographic regions (Öland and mainland), among populations within regions, and among individuals within populations was estimated by partitioning the sampled localities as Swedish mainland or Öland, followed by the analysis of molecular variance (AMOVA) procedure in ARLEQUIN using 1,564 AFLP loci for 20 populations of *Tetrix subulata*

Region	Source of variation	*df*	Sum of squares	Variance components	Percentage of total variation	Fixation indexes	*p*
Öland/mainland	Among regions	1	2254.63	11.11	4.72	*F*_CT_: 0.0472	.002 ± .0013
	Among populations within regions	18	8898.76	16.98	7.20	*F*_SC_: 0.0756	<.001
	Within populations	323	67055	207.6	88.08	*F*_ST_: 0.1192	<.001
	Total	342	78208.4	235.7			

**Figure 5:**
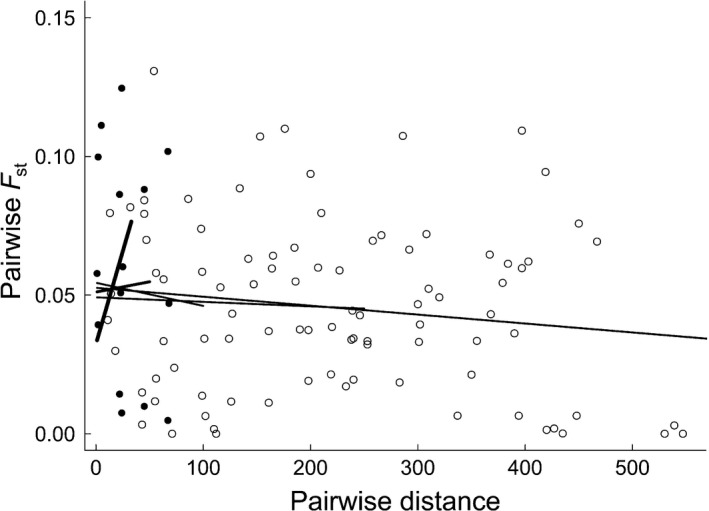
Pairwise genetic differences (estimated by pairwise *F*_ST_ on 1,564 AFLP loci) among 20 populations of pygmy grasshoppers *Tetrix subulata* were not correlated with the geographic distance (km) separating the sampling locations. Comparisons between locations on the Swedish mainland are indicated with open circles, and comparisons between locations on the island of Öland are indicated with black dots. On the mainland, a positive *F*_ST_–distance correlation was evident over short intervals, but the signature of isolation by distance disappeared over longer interpopulation distance intervals. The five regression lines represent relationships at five different interpopulation intervals (0–30, 0–50, 0–100, 0–250, and 0–550 km). The length of the regression lines (along the horizontal axis) correspond to the different interpopulation distances

There was no clear signature of isolation by distance among populations within geographic regions. Genetic distances between pairs of populations estimated by Slatkin's linearized *F*_ST_ were not positively correlated with the geographic distances separating populations (Mantel test, result for pooled regions: *r *=* *−.24, *p *=* *.015; Swedish mainland: *r *=* *−.27, *p *=* *.026; Öland: *r *=* *−.26, *p *=* *.133, Figure 5). Although the *F*_ST_–distance correlation was absent (or even negative) when calculated over all mainland sites, correlations calculated over more closely located mainland sites (<50 km) were clearly positive (Figure 5).

Pairwise *F*_ST_ values calculated between two small populations or between one small and one large population (average *F*_ST_ = 0.062, *n *=* *112) were not lower than estimates based on comparisons between two large populations (average *F*_ST_ = 0.058, *n *=* *78, ANOVA, *F*_1,188_ = 0.55, *p *=* *.46), indicating that the results of our analyses of population differentiation were not influenced by population size.

Estimated gene flow (*Nm*) indicated that migration rate between populations (mean = 8) ranged from 1.7 to 2,500 individuals per generation (Table 2). Estimated gene flow was highest by far between populations A25 and A28, despite that this was one of the most separated (ca 430 km) population pairs (Figure 2).

### 3.3 Association of genetic diversity with estimates of population size and immigration rate

**Figure 6:**
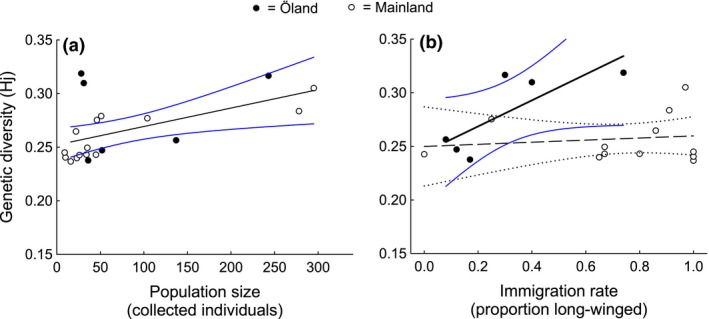
Relationships of genetic diversity (Hj) in populations of *Tetrix subulata* pygmy grasshoppers with (a) population size (as estimated by number of collected individuals) and (b) immigration rate (as estimated by proportion of long‐winged flight‐capable phenotypes). Data for populations on the Swedish mainland (open circles) and the island of Öland (filled dots). Figure shows least‐squares linear regression lines and 95% CI. The relationship linking genetic diversity to immigration rate depended on geographic region and was significant in the insular populations (solid black and blue lines) but not in the mainland (dashed and dotted black lines) populations (see text and Table 4)

Much (83%) of the total variation among populations in the level of within‐population genetic diversity, estimated by Hj, could be accounted for by geographic region together with our estimates of population size and immigration rate (Table 4). As expected, intrapopulation genetic diversity increased with increasing population size (Table 4, Figure 6a). Results regarding the positive association of genetic diversity (Hj) with population size remained qualitatively unchanged when number of grasshoppers collected per person (to control for differences in search effort) was used as a proxy for population size (cf Tables 4 and 5).

**Table 4:** Results from general linear model analysis of variance (GLM) for effects of geographic region (mainland vs. island), immigration (estimated by proportion of long‐winged individuals), and population size (estimated by number of individuals collected per visit), respectively, on genetic diversity (Hj, as estimated based on data for 1,564 AFLP loci) within 20 populations of *Tetrix subulata* pygmy grasshoppers. *df* represents nominator and denominator degrees of freedom. Eta‐squared, η^2^, is a measure of local effect size (Cohen, 1988). *F*‐values represent Type III tests for fixed effects. The overall model was significant (*F*_4,14_ = 16.76, *p *<* *.0001, *R* ^*2*^ = .83). Interactions that were not statistically significant (all *p *>* *.10) were removed from the model. *p*‐values in boldface are statistically significant

Source of variation	*df*	Estimate ± SE	η^2^	*F*	*p*
Geographic region	1,14	−0.026 ± 0.143	0.04	3.34	.0892
Population size	1,14	0.00020 ± 0.000036	0.37	24.90	**<.0001**
Proportion of long‐winged	1,14	−0.0062 ± 0.0131	0.29	23.33	**.0003**
Region by long‐winged interaction	1,14	0.147 ± 0.0286	0.33	26.37	**.0001**
Mainland region only					
Population size	1,10	0.00019 ± 0.000038	0.71	26.07	**.0005**
Proportion of long‐winged	1,10	−0.0056 ± 0.0119	0.01	0.22	.6474
Öland region only					
Population size	1,3	0.00022 ± 0.000102	0.23	4.74	.1176
Proportion of long‐winged	1,3	0.143 ± 0.0356	0.80	16.13	**.0277**

**Table 5:** Results from general linear model analysis of variance (GLM) for effects of geographic region (mainland vs. island), immigration (estimated by proportion of long‐winged individuals), and population size (estimated by number of individuals collected per person and visit), respectively, on genetic diversity (Hj, as estimated based on data for 1,564 AFLP loci) within 20 populations of *Tetrix subulata* pygmy grasshoppers. *df* represents nominator and denominator degrees of freedom. Eta‐squared, η^2^, is a measure of local effect size (Cohen, 1988). *F*‐values represent Type III tests for fixed effects. The overall model was significant (*F*_4,13_ = 18.77, *p *<* *.0001, *R* ^*2*^ = .85). Interactions that were not statistically significant (all *p *>* *.10) were removed from the model. *p*‐values in boldface are statistically significant

Source of variation	*df*	Estimate ± SE	η^2^	*F*	*p*
Geographic region	1,13	−0.025 ± 0.013	0.05	3.94	.0690
Population size	1,13	0.00089 ± 0.000140	0.46	40.61	**<.0001**
Proportion of long‐winged	1,13	−0.0061 ± 0.0116	0.29	25.18	**.0002**
Region by long‐winged interaction	1,13	0.138 ± 0.0257	0.33	28.98	**.0001**
Mainland region only					
Population size	1,10	0.00083 ± 0.000161	0.71	26.68	**.0004**
Proportion of long‐winged	1,10	−0.0508 ± 0.0118	0.005	0.19	.6758
Öland region only					
Population size	1,2	0.00108 ± 0.000306	0.33	12.53	.0714
Proportion of long‐winged	1,2	0.136 ± 0.0245	0.82	30.82	**.0310**

The effect on genetic diversity of immigration rate, as estimated by the incidence of the long‐winged phenotype, depended on region (Table 4). The significant interaction reflected that genetic diversity increased with increasing proportion of long‐winged phenotypes in populations on Öland, but not in populations on the mainland (Table 4, Figure 6b). The conclusion that there was a positive effect of immigration on genetic diversity on Öland but not on the mainland remained unchanged also when the more isolated mainland populations (A21, A22, A23, A24, A25, A28, A29, and A33, see Figure 2) were excluded from the comparison (effect of the interaction between region and proportion of long‐winged individuals: *F*_1,6_ = 24.46, *p *=* *.0026).

## 4 Discussion

Our analyses of 1,564 polymorphic AFLP markers demonstrate low to moderate genetic diversity (PPL = 59.5–90.1; Hj = 0.24–0.32) within and significant differentiation among (overall *F*_ST_ value of 0.25) 20 *Tetrix subulata* pygmy grasshopper populations sampled in southern Sweden. The signature of divergence was particularly strong between populations from the mainland and populations from the island of Öland situated 5 km off the Swedish east coast in the Baltic Sea, but significant differentiation was evident also within the two regions. The overall genetic structure seen in these grasshoppers can be accounted for by a combination of landscape structure and habitat configuration, together with effects of immigration and (re)colonizations on gene flow, and effects of drift associated with small population sizes, as discussed below.

### 4.1 Associations of genetic diversity with population size and immigration

The populations included in this study varied in size, interpopulation distances, and connectedness and were selected to evaluate whether and how drift, gene flow, and founder events influenced diversity within subpopulations. We found that estimates of within‐population genetic diversity (Hj) increased overall with increasing population size. This suggests that rare alleles may have been lost from smaller populations due to the eroding effects of random genetic drift, in concordance with predictions from neutral genetic theory (Crow & Kimura, 1970; Wright, 1931). The positive association between genetic diversity and population size seen in these grasshoppers conforms to the pattern reported in comparisons across species in different types of organisms (Frankham, 1996, 2012; Mager et al., 2014; Soulé & Wilcox, 1980).

Such within‐species studies typically evaluate the consequences of gene flow by analyzing genetic differentiation between populations in relation to geographic distance and potential dispersal barriers. The impacts of migration, (re)colonizations, and admixture on genetic diversity within subpopulations have been less investigated. We found that the level of genetic diversity within populations increased with increasing proportion of long‐winged phenotypes across populations on Öland, but no such association was evident on the Swedish mainland. Importantly, this differential pattern remained unchanged when the most isolated populations on the mainland were excluded from the analyses such that the comparison was made among mainland and insular groups of populations separated by comparable geographic distances. These results thus seem to indicate that the positive effects of gene flow from immigration and admixture on diversity were stronger on Öland. Previous studies show that *T. subulata* lead a sedentary lifestyle, although macropterous individuals have the capacity to disperse longer distances (Berggren et al., 2012). It is probably mainly macropterous individuals that contribute to dispersal and generate gene flow. An explanation for the stronger signature of immigration among populations on Öland may be that the Öland landscape is strongly fragmented and influenced by agriculture. The areas of suitable pygmy grasshopper habitat are patchily distributed, like islands surrounded by a “sea” of barren farmland used for growing crops that cannot harbor stable populations and that may hinder dispersal and gene flow. Wings might therefore be more important for gene flow on Öland than in the southern part of the Swedish mainland, where the landscape is more diversified.

That the level of genetic diversity within populations was not associated with the incidence of long‐winged phenotypes across mainland populations might reflect the combined outcome of different processes (immigration and admixture vs. founder events) with opposing effects on genetic diversity.

It is less likely that the relationship between genetic diversity and long‐winged phenotypes on Öland was influenced to any important degree by founder events, because one would normally hypothesize populations that have been recently established by a few founder individuals to have lower (not higher) genetic diversity (Frankham, Ballou, & Briscoe, 2010; Simberloff, 2009). However, there is firm experimental evidence from a diversity of organisms (including pygmy grasshoppers) that greater levels of genetic and phenotypic diversity promote establishment success (Forsman, 2014). Accordingly, populations resulting from colonization events by founder groups characterized by low genetic diversity can be expected to have low persistence. Such populations (with a high incidence of long‐winged individuals and low diversity) would therefore have been underrepresented in our data set. Furthermore, the production of half‐sibling offspring by polyandrous pygmy grasshopper females mated with several males (Caesar et al., 2007; Johansson et al., 2013) might have countered the expected genetic footprints of founder events (Pearse & Anderson, 2009).

Genetic diversity is essential for population viability and adaptation to changing environments (Hedrick, 2001; Reed & Frankham, 2003). Our present findings thus reinforce the notion that population size and immigration are key aspects for successful conservation of natural populations. It should be emphasized here that within the context of population and conservation genetics, it is generally effective population size, which can be complicated to accurately measure, that is of prime concern (Lande & Barrowclough, 1987). Our results suggest that, in some cases, even easily obtained estimates of census population size can inform about the level of genetic diversity, and hence presumably about evolutionary potential.

### 4.2 Interpopulation genetic differentiation

An important issue in population genetics and conservation biology is the degree to which landscape features and human land use create barriers to gene flow and lead to discrete population structure. Pygmy grasshoppers were sampled from the southern part of the Swedish mainland and from a large island (Öland) in the Baltic proper. The open water separating Öland from the mainland was hypothesized to act as a dispersal barrier, reducing gene flow and contributing to large‐scale genetic differentiation. That some of the mainland populations that were close to the island (A57 and A58) grouped with the Öland populations along axis 2 in Figure 4 was inconsistent with this hypothesis. However, other mainland populations (A20 and A31) that were also close to the island did not group with the Öland populations, and our overall findings were in agreement with the prediction that the open water restricted migration and gene flow. Results from the AMOVA showed significant differentiation between the two geographic regions, and this differentiation was evident when only the six mainland populations closest to the island of Öland were included in the comparison. This large‐scale structure and separation between grasshoppers on Öland and the Swedish mainland were further supported by the Bayesian cluster analysis, which indicated that the 20 populations of *T. subulata* were clustered into three groups, two of which were located on Öland.

The two clusters on Öland might represent different colonization events separated in time or descendants to colonizers that originated from geographically and genetically different source populations. That populations belonging to the two different clusters on Öland existed relatively close to each other might be due to behavioral reproductive isolation or reflect negative fitness effects of interpopulation hybridization and genetic admixture (Tinnert, Berggren, & Forsman, 2016). It is important to determine whether they belong to one recombining population, or whether they are ecologically and reproductively isolated members of two evolutionarily significant units (Hey, Waples, Arnold, Butlin, & Harrison, 2003). If individuals recombine freely at sites of coexistence, then the clustering may be just a ghost of history, of limited importance for the evolutionary dynamics of the species at present.

Results from separate analyses of molecular diversity on the mainland and island, respectively, revealed moderate pairwise genetic distances (overall *F*_ST_ –values of 0.25) indicating genetic divergence among populations from different sampling localities within each of the two regions. Subpopulations are generally considered to be greatly genetically differentiated if they exhibit differentiation indexes (*F*_ST_) in the range of 0.25 (Wright, 1978). The nonpanmictic distribution shows that pygmy grasshoppers do not disperse and interbreed freely among localities. This conclusion conforms well with the finding in previous studies of local adaptive differentiation and of rapid evolutionary shifts in functionally important traits in response to divergent and fluctuating selection (Forsman et al., 2011, 2012; Tinnert et al., 2016; Wennersten et al., 2012). That Orthopterans can exhibit deep genetic differentiation at fine geographical scales has been demonstrated also in other species of grasshoppers, such as *Mioscirtus wagneri* on the Iberian peninsula (Ortego, Aguirre, & Cordero, 2010).

### 4.3 Isolation by distance—depending on distance

The level of genetic differentiation between populations is expected to decrease with decreasing geographic distance under the hypothesis that gene flow has a diluting effect (Slatkin, 1993; Wright, 1943). We found no evidence for isolation by distance among our populations despite that we performed separate analyses for mainland and insular populations. Such lack of isolation by distance could be interpreted as a complete lack of gene flow or indicate extensive gene flow among subpopulations due to the lack of any distance‐related or physical barriers to dispersal (van Strien et al., 2015). To discriminate between these competing explanations and further assess the presence and intensity of any isolation by distance, we evaluated the *F*_ST_–geographic distance correlation from subsets of mainland population pairs that differed with regard to threshold interpopulation distance (van Strien et al., 2015). This showed that the *F*_ST_–distance correlation was absent when calculated over all mainland sites, whereas correlations calculated over more closely located mainland sites (<50 km) were clearly positive (Figure 5).

These results underscore the importance of taking into consideration the geographical structure and scale of sampling when evaluating consequences of immigration, and further illustrate that caution is needed when interpreting results from isolation‐by‐distance tests in studies of population genetic structure (Hutchison & Templeton, 1999; Rousset, 1997; van Strien et al., 2015). Dispersal may influence genetic diversity within populations and contribute to patterns of divergence among populations even if there is no clear signal of separation by distance (Merimans, 2012). Reasons for this include nonrandom genotype‐dependent dispersal (Berggren et al., 2012; Edelaar et al., 2008) and condition‐dependent or population‐specific effects of admixture (Rius & Darling, 2014; Tinnert et al., 2016). Furthermore, in studies with a nonexhaustive sampling scheme, the contribution of immigrants from source populations that were not sampled may be underestimated (Merimans, 2012; Yang et al., 2012). The sampling scheme dilemma is likely more pronounced in homogeneous environments than in patchy and sharp transition landscapes (e.g., islands or lakes), but, to our knowledge, this has not yet been investigated.

That the degree of genetic differentiation among subpopulations was only weakly related to geographic isolation, and only over smaller spatial scales, in the present study may be attributed in part to the ecological characteristics and population dynamics of our study species. Pygmy grasshoppers are environmental trackers that can establish and flourish for a few years when and where conditions are favorable, typically in disturbed environments (Berggren et al., 2012; Forsman et al., 2011). Such abundance fluctuations and extinction (re)colonization dynamics, combined with that they occur at low density over a broad range of mainland habitats, could have a homogenizing effect on neutral population genetic structure (Frankham, 1996; Lande, 1988).

An additional potential explanation for the weak signature of isolation by distance might be related to the spatial arrangement of particular study populations, because the populations from the northern, western, and southern outskirts of the mainland sampling area were relatively small (Table 1, Figure 2). Previous studies suggest that population size can influence the genetic distance between populations (e.g., Mager et al., 2014). However, pairwise *F*_ST_ values in the present study were not smaller overall in comparisons that involved relatively small populations, indicating that our estimates of genetic differentiation were not influenced to any important degree by stochastic effects associated with small population sizes.

## 5 Summary and Conclusions

Our data on AFLP markers demonstrate low to moderate genetic diversity within and significant divergence among populations of pygmy grasshoppers from 20 sampling localities in southern Sweden. Genetic diversity increased with increasing population size and with increasing proportion of long‐winged phenotypes across population on the island of Öland, thus implicating immigration as an important determinant of within‐population diversity. That no association with immigration was evident on the Swedish mainland could reflect differences in population dynamics, habitat heterogeneity, and connectedness between Öland and the mainland. Our data further suggested that the open water (5 km) that separates Öland from the Swedish mainland has restricted gene flow and leads to genetic divergence among geographic regions. The positive association between genetic divergence and geographic separation expected under the isolation‐by‐distance hypothesis was only evident over short interpopulation distances (<50 km) on the mainland and gradually disappeared as populations separated by longer distances (up to 550 km) were included.

In conclusion, this study provides an empirical example that integrating ecological and molecular data are key to identifying processes that influence population genetic structure and diversity in natural populations. Our results specifically demonstrate that even crude estimates of census population size can have high predictive power regarding the level of genetic diversity and evolutionary potential. Our findings also illustrate the potential of using the incidence of flight‐capable phenotypes as a proxy for immigration in investigations of genetic structure in wing‐polymorphic species. Finally, the overall results regarding population differentiation further underscore the importance of landscape structure and spatial sampling scheme for conclusions regarding the role of gene flow and isolation by distance.

## Conflict of Interest

None declared.

## Data Accessibility

Data on sampling locations, sex, wing morph, and AFLP genotypes of individuals used in the analyses will be made available at Dryad Digital Repository following acceptance of the manuscript.
