# Supplementary material for: Population genetic structure, differentiation, and diversity in Tetrix subulata pygmy grasshoppers: roles of population size and immigration
Source: Ecol Evol. 2016 Oct 9;6(21):7831–46. doi: 10.1002/ece3.2520 (PMC6093165; doi:10.1002/ece3.2520)
Supplement: Supplementary file 1 [file ECE3-6-7831-s001.docx]

**Population genetic structure, differentiation, and diversity in *Tetrix subulata* pygmy grasshoppers: roles of population size and immigration**

Jon Tinnert, Olof Hellgren, Jenny Lindberg, Per Koch-Schmidt, & Anders Forsman

***Supporting Information***

**Results based on a matrix of 638 (rather than 1564) AFLP loci**

**Population genetic variance within populations**

Analyses of the 638 polymorphic AFLP markers for 345 *Tetrix subulata* pygmy grasshopper individuals revealed high within population genetic diversity (proportion polymorphic loci and average heterozygosity) across all 20 sampling localities (PPL = 29.6 to 67.7; Hj = 0.12 to 0.21, **Table S1**).

**Population genetic structure**

The signature of genetic structure among populations of *T. subulata* was low to moderate, as indicated by significant pairwise *F*_ST_ values that ranged from 0 to 0.18 (AFLPsurv) (**Table S2)** by the likelihood values obtained in the assignment test (Structure) (**Fig S1**), and by results of the Principal Coordinate analysis (**Fig. S2**).

Results of the nested AMOVA provided weak but statistically significant evidence for spatial population structure (**Table S3**). Nesting sample location by geographic region accounted for 3.8% of the total variance while 6.3% was explained by variation among sample locations within geographic regions, and 89.9% was explained by variation among individuals within sample locations.

**Isolation by distance**

Genetic differentiation among populations, estimated by Slatkin’s linerarized *F*_ST_, was not significantly correlated with the geographic distance separating populations (Mantel test, results for pooled regions: *r* = -0.095, *p* = 0.228; Swedish mainland: *r* = -0.10, *p* = 0.34; Öland: *r* = -0.28, *p* = 0.079). On the mainland, a positive *F*_ST_-distance correlation was evident over short intervals, but the signature of isolation by distance disappeared over longer inter-population distance intervals (**Fig. S3**).

**Association of genetic diversity with estimates of population size and immigration**

Much (75%) of the total variation among populations in the level of within population genetic diversity, estimated by Hj, could be accounted for by geographic region together with our estimates of population size and immigration rate (**Table S4**). As expected, intra population genetic diversity increased with increasing population size (**Table S4**).

The effect on genetic diversity of immigration rate, as estimated by the incidence of the long-winged phenotype, depended on region (**Table S4**). The significant interaction reflected that genetic diversity increased with increasing proportion long-winged phenotypes in populations on Öland, but not in populations on the mainland (**Table S4**).

**Table S1.** Genetic diversity in samples of *Tetrix subulata* pygmy grasshoppers collected from 20 sampling locations on the Swedish mainland and on the island of Öland off the Swedish east coast in the Baltic Sea. *N* indicates number of sampled individuals used for AFLP analyses, PL indicates number of polymorphic loci, PPL indicates percentage polymorphic loci, and Hj indicates genetic diversity. The dashed line marks the separation of sample sites from the Swedish mainland (above line) and Öland (below line).

| **Sample ID** | **Group** | **Sample name** | **Year** | ***N*** | **Habitat type** | **Sampled individuals** | **Wing freq.**  **(N)** | **Coordinates**  **Lat. Long.** | **PL** | **PPL** | **Hj (S.E.)** |
| --- | --- | --- | --- | --- | --- | --- | --- | --- | --- | --- | --- |
| A20 | Mainland | Eneskärskläppen | 2011 | 11 | Island vegetation | 16 | 1 (16) | 56.866850°,16.495717° | 189 | 29.6 | 0.11551 (0.00629) |
| A21 | Mainland | Edane | 2012 | 14 | Pasture | 22 | 0.86 (22) | 59.667833°,12.830050° | 236 | 37.0 | 0.15391 (0.00696) |
| A22 | Mainland | Tindered | 2012 | 9 | Pasture | 26 | 1 (10) | 57.977083°,16.484550° | 294 | 46.1 | 0.13654 (0.00674) |
| A23 | Mainland | Knutby | 2012 | 21 | Pasture | 23 | 0.65 (20) | 59.909033°,18.290467° | 252 | 39.5 | 0.11545 (0.00609) |
| A24 | Mainland | Hyllinge | 2012 | 22 | Pond | 35 | 0.67 (35) | 56.103217°,12.895167° | 229 | 35.9 | 0.12640 (0.00627) |
| A25 | Mainland | Sjöbo | 2012 | 25 | Pasture | 45 | 0.67 (45) | 55.648617°,13.694067° | 227 | 35.6 | 0.11557 (0.00598) |
| A28 | Mainland | Gnesta | 2012 | 24 | Wet agricultural area | 34 | 0.8 (30) | 59.029900°,17.335900° | 224 | 35.1 | 0.11798 (0.00617) |
| A29 | Mainland | Simrishamn | 2012 | 26 | Pasture | 26 | 0 (24) | 55.549767°,14.351933° | 229 | 35.9 | 0.11818 (0.00609) |
| A30 | Mainland | Tomtesunda | 2012 | 10 | Pasture | 26 | 1 (9) | 56.173883°,15.479167° | 307 | 48.1 | 0.13580 (0.00661) |
| A31 | Mainland | Ålem | 2009 | 16 | Agricultural area | 278 | 0.91 (11) | 56.933650°,16.363467° | 264 | 41.4 | 0.14796 (0.00673) |
| A33 | Mainland | Hägern | 2009 | 15 | Meadow nearby burnt area. | 104 | 0.92 (12) | 57.423067°,16.266067° | 253 | 39.7 | 0.14575 (0.00661) |
| A45 | Mainland | Sävsjö | 2011 | 22 | Pasture with pond | 295 | 0.97 (198) | 56.537800°,15.803867° | 405 | 63.5 | 0.18915 (0.00676) |
| A57 | Mainland | Aspelund | 2008 | 4 | Pasture with stream | 46 | 0.25 (4) | 56.553767°,16.022617° | 227 | 35.6 | 0.16447 (0.00743) |
| A58 | Mainland | Björnö | 2008 | 9 | Pasture | 51 |  | 56.770617°,16.364550° | 324 | 50.8 | 0.15113 (0.00697) |
| A37 | Öland | Bredsätra | 2011 | 24 | Pasture with pond | 52 | 0.12 (41) | 56.849283°,16.788883° | 220 | 34.5 | 0.14106 (0.00701) |
| A44 | Öland | Vanserumbäck | 2011 | 19 | Pasture with stream | 243 | 0.3 (200) | 56.673733°,16.636900° | 432 | 67.7 | 0.20535 (0.00659) |
| A49 | Öland | Jordtorp | 2011 | 21 | Pasture and alkaline fen | 36 | 0.17 (30) | 56.676883°,16.555583° | 254 | 39.8 | 0.13326 (0.00683) |
| A54 | Öland | Norra mossen | 2007 | 17 | Pasture | 31 | 0.4 (25) | 56.861850°,16.779167° | 427 | 66.9 | 0.21127 (0.00642) |
| A60 | Öland | Hörninge | 2008 | 16 | Clear-cut with alkaline fen | 28 | 0.74 (27) | 56.858100°,16.768433° | 410 | 64.3 | 0.21516 (0.00662) |
| A63 | Öland | Lindby | 2010 | 20 | Alkaline grassland with stream | 137 | 0.08 (65) | 56.280283°,16.456100° | 292 | 45.8 | 0.14528 (0.00667) |
|  |  | Total |  | 345 |  | 1548 |  |  | 638 |  | 0.31 (0.025) |

**Table S2**. Population structure estimated in AFLPsurv with 638 loci on 20 populations of *Tetrix subulata.* Lower matrix indicates F_ST_ values; upper matrix indicates *Nm* values. See Table 1 for a key to abbreviations of sampling locations. The dashed lines separate locations on the Swedish mainland from locations on Öland.

|  | A20 | A21 | A22 | A23 | A24 | A25 | A28 | A29 | A30 | A31 | A33 | A45 | A57 | A58 | A37 | A44 | A49 | A54 | A60 | A63 |
| --- | --- | --- | --- | --- | --- | --- | --- | --- | --- | --- | --- | --- | --- | --- | --- | --- | --- | --- | --- | --- |
| A20 |  | 2.12 | 5.7 | 9.11 | 7.69 | 6.16 | 6.02 | 4.98 | 5.72 | 5.37 | 7.44 | 3.4 | 1.48 | 3.04 | 2.11 | 2.66 | 2.32 | 1.6 | 1.78 | 2.52 |
| A21 | 0.1053 |  | 10.62 | 1.67 | 2.03 | 1.56 | 1.78 | 1.71 | 2.19 | 3.05 | 3.04 | 6.28 | 80.4 | 3.31 | 6.68 | 4.77 | 3.54 | 5.79 | 4.35 | 3.36 |
| A22 | 0.042 | 0.023 |  | 3.04 | 3.57 | 2.62 | 3.54 | 2.8 | 3.52 | 3.86 | 4.31 | 5.28 | 5.22 | 2.51 | 4.77 | 3.55 | 4.21 | 3.18 | 2.65 | 4.29 |
| A23 | 0.0267 | 0.1305 | 0.0759 |  | 833.08 | - | - | 18 | 227.02 | 18.27 | 9.75 | 3.43 | 1.14 | 3.99 | 1.94 | 2.49 | 2.29 | 1.46 | 1.73 | 2.66 |
| A24 | 0.0315 | 0.1097 | 0.0654 | 0.0003 |  | - | 178.32 | 49.75 | 124.75 | 20.94 | 12.64 | 4.18 | 1.3 | 4.6 | 2.29 | 2.84 | 2.63 | 1.62 | 2.07 | 3.23 |
| A25 | 0.039 | 0.1385 | 0.0872 | 0 | 0 |  | 499.75 | 42.12 | 118.8 | 11.49 | 10.67 | 3.36 | 1.1 | 3.96 | 1.86 | 2.37 | 2.23 | 1.41 | 1.76 | 2.63 |
| A28 | 0.0399 | 0.1231 | 0.0659 | 0 | 0.0014 | 0.0005 |  | 34.47 | - | 14.28 | 10.81 | 3.65 | 1.15 | 4.03 | 2.11 | 2.66 | 2.5 | 1.57 | 1.9 | 3.16 |
| A29 | 0.0478 | 0.1275 | 0.082 | 0.0137 | 0.005 | 0.0059 | 0.0072 |  | 21.87 | 13.05 | 11.54 | 3.65 | 1.16 | 4.31 | 1.96 | 2.68 | 2.17 | 1.51 | 1.92 | 2.66 |
| A30 | 0.0419 | 0.1023 | 0.0663 | 0.0011 | 0.002 | 0.0021 | 0 | 0.0113 |  | 11.43 | 10.3 | 4.48 | 1.42 | 4.74 | 2.65 | 3.12 | 3 | 1.84 | 2.17 | 3.63 |
| A31 | 0.0445 | 0.0758 | 0.0608 | 0.0135 | 0.0118 | 0.0213 | 0.0172 | 0.0188 | 0.0214 |  | 40.73 | 11.77 | 1.88 | 25.26 | 3.33 | 5.5 | 3.48 | 2.6 | 3.67 | 4.48 |
| A33 | 0.0325 | 0.0759 | 0.0548 | 0.025 | 0.0194 | 0.0229 | 0.0226 | 0.0212 | 0.0237 | 0.0061 |  | 20.08 | 1.98 | 18.41 | 3.07 | 7.71 | 2.8 | 2.64 | 3.74 | 4.18 |
| A45 | 0.0684 | 0.0383 | 0.0452 | 0.068 | 0.0564 | 0.0693 | 0.0641 | 0.0641 | 0.0528 | 0.0208 | 0.0123 |  | 4.04 | 30.61 | 4.59 | - | 3.11 | 5.36 | 12.98 | 4.46 |
| A57 | 0.1444 | 0.0031 | 0.0457 | 0.1798 | 0.1608 | 0.1846 | 0.1782 | 0.1777 | 0.1497 | 0.1175 | 0.1123 | 0.0583 |  | 1.86 | 7.1 | 3.56 | 3.74 | 12.64 | 2.94 | 3.28 |
| A58 | 0.0761 | 0.0703 | 0.0907 | 0.059 | 0.0515 | 0.0594 | 0.0584 | 0.0548 | 0.0501 | 0.0098 | 0.0134 | 0.0081 | 0.1186 |  | 2.57 | 11.11 | 2.03 | 2.36 | 8.55 | 2.86 |
| A37 | 0.106 | 0.0361 | 0.0498 | 0.1142 | 0.0984 | 0.1187 | 0.1059 | 0.1132 | 0.0861 | 0.0698 | 0.0753 | 0.0516 | 0.034 | 0.0888 |  | 3.44 | 31.4 | 6.85 | 2.65 | 23.56 |
| A44 | 0.0859 | 0.0498 | 0.0658 | 0.0914 | 0.0808 | 0.0954 | 0.086 | 0.0853 | 0.0742 | 0.0435 | 0.0314 | 0 | 0.0656 | 0.022 | 0.0678 |  | 2.42 | 5.01 | 25.52 | 3.2 |
| A49 | 0.0971 | 0.0659 | 0.056 | 0.0985 | 0.0867 | 0.101 | 0.0909 | 0.1032 | 0.077 | 0.0671 | 0.082 | 0.0743 | 0.0627 | 0.1098 | 0.0079 | 0.0937 |  | 4.26 | 2 | 25.79 |
| A54 | 0.135 | 0.0414 | 0.0729 | 0.1462 | 0.1335 | 0.1505 | 0.1371 | 0.1422 | 0.1194 | 0.0878 | 0.0866 | 0.0446 | 0.0194 | 0.0958 | 0.0352 | 0.0475 | 0.0554 |  | 4.33 | 4.9 |
| A60 | 0.123 | 0.0543 | 0.0862 | 0.1265 | 0.1076 | 0.1246 | 0.1165 | 0.1154 | 0.1034 | 0.0638 | 0.0627 | 0.0189 | 0.0783 | 0.0284 | 0.0863 | 0.0097 | 0.1113 | 0.0546 |  | 2.52 |
| A63 | 0.0903 | 0.0693 | 0.0551 | 0.086 | 0.0719 | 0.0867 | 0.0734 | 0.086 | 0.0644 | 0.0528 | 0.0564 | 0.0531 | 0.0709 | 0.0803 | 0.0105 | 0.0724 | 0.0096 | 0.0485 | 0.0902 |  |

**Table S3**. Genetic variance among geographic regions (Öland and mainland), among populations within regions, and among individuals within populations was estimated by partitioning the sampled localities as Swedish mainland or Öland, followed by the analysis of molecular variance (AMOVA) procedure in Arlequin using 638 AFLP loci on 20 populations of *Tetrix subulata*.

| Region | Source of variation | d.f. | Sum of squares | Variance components | Percentage of total variation | Fixation indexes | *P* |
| --- | --- | --- | --- | --- | --- | --- | --- |
| Öland/mainland | Among regions | 1 | 423.06 | 2.01 | 3.83 | FCT: 0.038 | 0.003 ± 0.0016 |
|  | Among populations within regions | 18 | 1857.99 | 3.29 | 6.26 | FSC: 0.065 | < 0.001 |
|  | Within populations | 325 | 15369.17 | 47.29 | 89.92 | FST: 0.101 | < 0.001 |
|  | Total | 344 | 17650.21 | 52.59 |  |  |  |

**Table S4.** Results from general linear model analysis of variance (GLM) for effects of geographic region (mainland *versus* island), immigration (as estimated by proportion long-winged individuals) and population size (as estimated by number of individuals collected per visit), respectively, on estimates of genetic diversity (Hj, as estimated based on data for 638 AFLP loci) within 20 populations of *Tetrix subulata* pygmy grasshoppers. *df* represents nominator and denominator degrees of freedom. Eta-squared, *η*^2^, is a measure of local effect size ([Cohen 1988](#_ENREF_1)). F represents *F*-value for Type III tests for fixed effects. The overall model was significant (*F*_4,14_ = 10.75, *P* = 0.0003, *R^2^* = 0.75). Interactions that were not statistically significant (all *P* > 0.10) were removed from the model.

| Source of variation | *df* | estimate ± SE | *η*^2^ | *F* | *P* |
| --- | --- | --- | --- | --- | --- |
| Geographic region | 1,14 | -0.009 ± 0.0197 | 0.003 | 0.19 | 0.6677 |
| Proportion long-winged | 1,14 | -0.0010 ± 0.0180 | 0.26 | 14.58 | **0.0019** |
| Population size | 1,14 | 0.00016 ± 0.00005 | 0.18 | 10.44 | **0.0060** |
| Region by long-winged interaction | 1,14 | 0.147 ± 0.0392 | 0.25 | 14.11 | **0.0021** |
|  |  |  |  |  |  |
| *Mainland region only* |  |  |  |  |  |
| Proportion long-winged | 1,10 | -0.0006 ± 0.01807 | 0.0001 | 0.00 | 0.9757 |
| Population size | 1,10 | 0.00016 ± 0.000058 | 0.41 | 7.39 | **0.0216** |
|  |  |  |  |  |  |
| *Öland region only* |  |  |  |  |  |
| Proportion long-winged | 1,3 | 0.148 ± 0.0411 | 0.80 | 12.95 | **0.0368** |
| Population size | 1,3 | 0.00018 ± 0.000117 | 0.14 | 2.33 | 0.2244 |

**
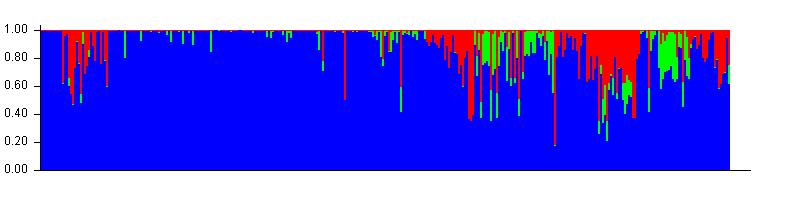

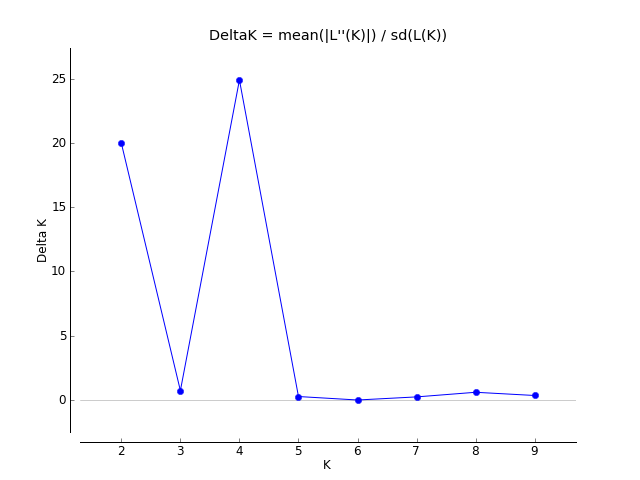

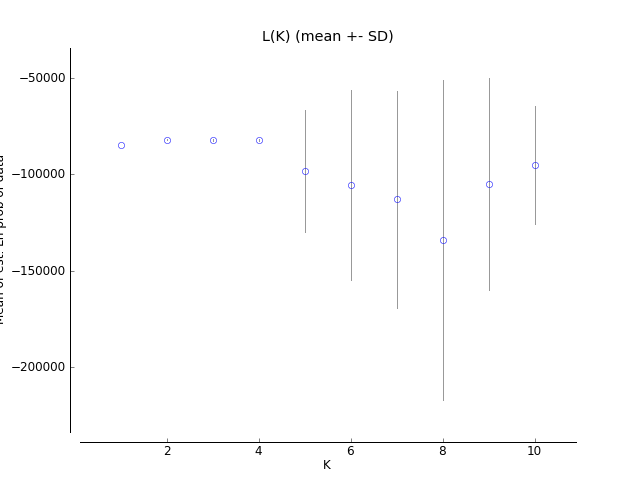
Figure S1**. Bayesian population structure analysis used to assign 345 *Tetrix subulata* individuals to different genetic clusters for AFLP genotypes. (A) Mean likelihood of 15 simulations of 1-10 populations (K) of pygmy grasshoppers. (B) Change (delta K) in likelihood for K=1-10 (Evanno et al. 2005). (C) Individual probability assignment of each of the individuals sampled in the 20 different locations for k=3 populations. The y-axis denotes the cumulative posterior probability of an individual’s placement in particular population(s). Data based on 638 variable AFLP markers. See Table S1 for a key to abbreviations of sampling localities.


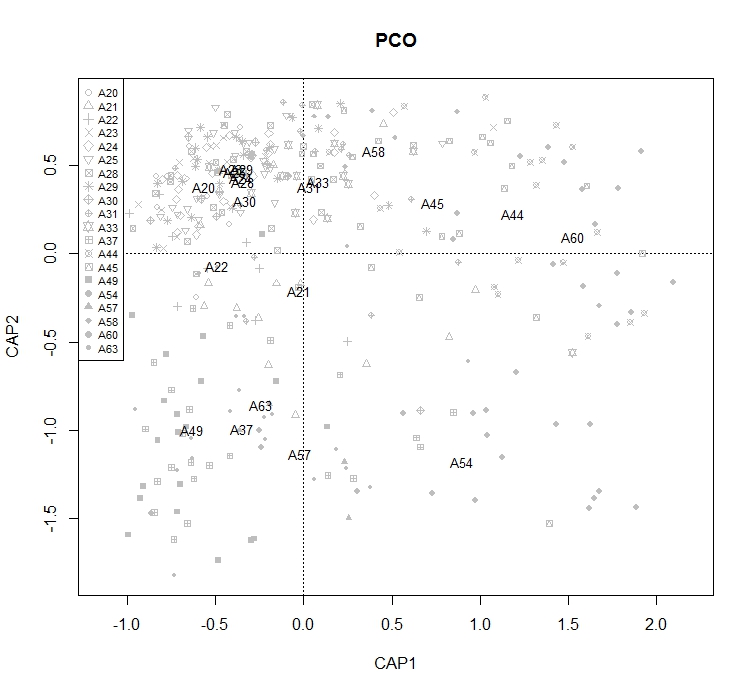


**Figure S2**. Principal Coordination analysis plots for pygmy grasshopper *Tetrix subulata* individuals collected at 20 sampling locations for 638 AFLP markers calculated using Jaccard distance. Axis 1 (horizontal) accounted for 32.2% of the total variation and axis 2 (vertical) accounted for 23.8 % of the variation.

**
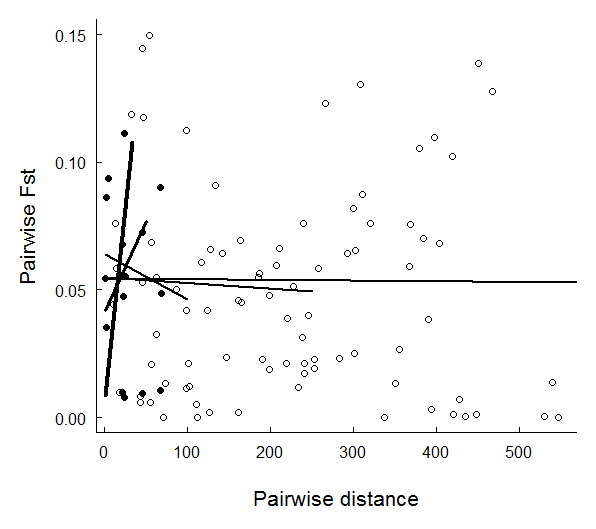
**

**Figure S3.** Pairwise genetic differences (estimated by pairwise *F*_ST_ on 638 AFLP loci) among 20 populations of pygmy grasshoppers *Tetrix subulata* were not correlated with the geographic distance (km) separating the sampling locations. Comparisons between locations on the Swedish mainland are indicated with open circles and comparisons between locations on the island of Öland are indicated with black dots. On the mainland a positive *F*_ST_-distance correlation was evident over short intervals, but the signature of isolation by distance disappeared over longer inter-population distance intervals. The different lines represent relationships at five different inter-population intervals (0-30km, 0-50 km, 0-100 km, 0-250 km, 0-550 km).

Cohen JE (1988) *Statistical power analysis for behavioral sciences* Lawrence Erlbaum Associates, Inc., Hillsdale, NJ.
